# Cyclic Vomiting Syndrome: A Case Report and Mini Literature Review

**DOI:** 10.7759/cureus.11695

**Published:** 2020-11-25

**Authors:** Tamadhir Al-Mahrouqi, Salim A Al Busaidi, Abdullah M Al Alawi

**Affiliations:** 1 Psychiatry, General Foundation Program, Oman Medical Specialty Board, Muscat, OMN; 2 Medicine, Sultan Qaboos University Hospital, Muscat, OMN

**Keywords:** nausea and vomiting, cyclic vomiting, episodic migraine, functional disorder

## Abstract

A 27-year-old man presented to the emergency department with nausea, vomiting, and abdominal pain. He had been having similar episodes for the last seven years, and all of the previous workups had been unremarkable. After excluding all organic causes of his presentation, the patient was diagnosed with cyclic vomiting syndrome (CVS) and managed accordingly. He has shown remarkable improvement and no further attack has been reported for almost five months since the diagnosis was made. This case report highlights the diagnostic challenge represented by CVS. Also, it summarizes the main aspects of management to achieve and maintain the remission of the condition.

## Introduction

Cyclic vomiting syndrome (CVS) is a chronic functional gastrointestinal disorder characterized by episodic nausea and vomiting. It is a well-known diagnosis in the pediatric population and has become more recognized in the adult population [1]. The exact pathogenesis of the condition is unknown, and several theories have been proposed. Migraine and CVS share similar pathophysiology, as suggested by several studies [2]. It is commonly misdiagnosed, and the delay in diagnosis is associated with significant morbidity and potential unnecessary intervention and surgeries [3]. Trigger avoidance, symptomatic treatment during the hyperemesis phase, and prophylactic medications are effective in reducing the severity and reoccurrence of this rare condition [4].

## Case presentation

A 27-year-old man presented to the emergency department with nausea and vomiting for a few days. The vomiting was non-projectile, non-bilious, and contained ingested food materials. The patient had no concomitant diarrhea and denied any constitutional symptoms.

The patient described experiencing similar episodes of vomiting over the last seven years. He reported that the typical episode started upon waking up in the morning with no preceding stressors and was associated with intense abdominal pain, lethargy, and nausea. Then, the abdominal pain would be followed by multiple episodes of vomiting up to 10 times within a few hours. Every episode might last for five to six days and would reoccur every two to three months. The patient was doing well between episodes and was able to resume his job as military personnel.

He had experienced many emergency presentations and multiple admissions and had been treated symptomatically throughout without reaching a conclusive diagnosis. His past medical history was unremarkable apart from a laparoscopic appendectomy performed seven years back.

The patient had achieved his developmental milestones at normal ages and had grown up in a middle-class family consisting of his parents, brothers, and sisters. He denied a previous diagnosis of any psychiatric disorders such as post-traumatic stress disorder. His siblings were healthy, except for his younger sister, who was known to have an intellectual disability. He was married and had a one-year-old child; he worked as a civil officer in the armed forces and denied alcohol consumption, smoking, or drug abuse. Clinical examination revealed normal vital signs, delayed capillary refill, and dry mucus membrane. The abdomen was soft, with mild epigastric tenderness. Other systemic examinations were unremarkable.

The patient was investigated thoroughly and evaluated for possible gastroenterological, immunological, neurological, metabolic, and psychiatric disorders. Biochemical investigations were normal, except for lactic acidosis, ketonuria, and proteinuria attributed to the normal physiological response to vomiting. Also, the urine toxicology screen was negative. An extensive gastrointestinal workup including abdominal X-ray, abdominopelvic CT, gastro-endoscopy, and colonoscopy was unremarkable. Neurologically, he was evaluated with brain MRI, magnetic resonance spectroscopy, and electroencephalography, and all were normal. The patient was also screened for various metabolic disorders, including urea cycle defect, porphyria and Fabry disease, organic aciduria, fatty acid oxidation disorder, and amino acid disorder; all of them were excluded. Other workups for the immunological and autoimmune disorders were unremarkable. He had undergone multiple psychiatric assessments during different admissions, which had been unable to establish any psychogenic cause of the vomiting.

After excluding all the above possibilities, we considered CVS, and the patient's presentation fulfilled the Rome IV criteria for the diagnosis of CVS. The patient was started on amitriptyline 25 mg (0.5 mg/kg) as a prophylactic measure. The patient responded to the treatment well; he has had a five-month symptom-free period and has remained well during outpatient follow-up visits.

## Discussion

CVS is a chronic functional gastrointestinal disorder characterized by episodic nausea and vomiting lasting for one to five days, followed by asymptomatic periods [3,5]. It is mainly a childhood disorder with an estimated prevalence between 0.04-1.9% but has become an increasingly recognized diagnosis in the adult population [3,6].

The exact pathophysiology of CVS is not known. Several potential pathophysiological factors have been suggested, including migraine-related mechanisms, mitochondrial DNA mutations, excessive hypothalamic-pituitary-adrenal axis activation, and autonomic dysfunction [2]. The prevalence of migraine headaches is common among patients with CVS, with an estimated prevalence of 36% and 46% in adult and pediatric patients, respectively [3,7]. Also, a family history of migraines is common among patients with CVS, which may suggest common pathophysiology [5]. Psychiatric comorbidities such as anxiety disorders, panic attacks, mood disorders, alcoholism, and drug abuse are common among patients with CVS [8,9].

In the adult population, CVS is characterized by severe episodes of emesis and severe abdominal pain [3,5]. Typically, it has four phases: prodromal, hyperemesis, recovery, and remission. The prodromal phase may last for minutes to several hours and usually consists of anorexia, abdominal pain, lethargy, pallor, and autonomic symptoms such as sweating [5,10]. The hyperemesis phase usually starts in the morning and is characterized by severe nausea, vomiting, and abdominal pain that lasts for several days (an average of 5.9 days). Nausea and vomiting decrease and appetite and energy improve during the recovery phase, followed by a symptom-free period (remission phase) [5]. The episodes can be triggered by menstrual cycle stress, infections, childbirth, sleep deprivation, certain foods, and alcohol or cannabis use [3,11,12].

CVS is commonly misdiagnosed as gastroesophageal reflux, food poisoning, gastroenteritis, conversion disorder, or eating disorders. The delay in diagnosis is more frequent in adults (7.9 years) compared to children (1.9 years) [3]. Also, undiagnosed patients may have severely impaired quality of life or maybe subjected to unnecessary surgeries and interventions [3,5].

The diagnosis of CVS should be made after excluding other causes of vomiting [4,5]. The Rome IV criteria have been used to diagnose CVS. It defines CVS as stereotypic episodes of vomiting with the following characteristics: 1) presence of at least two acute episodes of vomiting in the preceding six months, 2) each episode of vomiting persisting for less than one week, 3) at least one week of an interval between episodes of vomiting, and 4) an absence of vomiting between episodes [13].

The preventive strategy should be directed to identify and avoid triggers [5]. Antiemetics and intravenous (IV) hydration are the main treatment methods used for managing the acute phase of CVS. Also, benzodiazepine (for sedation and sleep induction) and opioids (for severe pain) may be used in the acute phase [5,10]. During remission, the goal of treatment should be to prevent further attacks [10]. Antidepressants (e.g., tricyclic antidepressants), antiepileptics (e.g., topiramate), and anti-migraines (e.g., sumatriptan) have been shown to lead to a significant reduction in relapses in the majority of cases [4,10].

Our patient had suffered from the condition for about seven years before he was diagnosed, which is consistent with the previously reported cases of CVS in adults. He responded very well to amitriptyline and has remained well during follow-up visits. We could increase the dose of amitriptyline to 50 mg (1 mg/kg) if he has further relapses.

## Conclusions

CVS is a rare condition with unclear pathophysiology. It may represent a diagnostic challenge for healthcare providers, and its delayed diagnosis is associated with considerable over-investigation, morbidity, social stress, and functional impairment in patients. After excluding organic causes of vomiting, the Rome IV criteria are an excellent tool to identify patients with CVS. Supportive measures, including antiemetics, IV hydration, sedation, and pain management, are the main aspects of CVS management. Several prophylactic drugs have been proven to be effective in reducing the frequency and severity of CVS in the majority of cases.

## References

[1] Bhandari S, Jha P, Thakur A, Kar A, Gerdes H, Venkatesan T (2018). Cyclic vomiting syndrome: epidemiology, diagnosis, and treatment. Clin Auton Res.

[2] Li BU, Misiewicz L (2003). Cyclic vomiting syndrome: a brain-gut disorder. Gastroenterol Clin North Am.

[3] Abell TL, Adams KA, Boles RG (2008). Cyclic vomiting syndrome in adults. Neurogastroenterol Motil.

[4] Venkatesan T, Levinthal DJ, Tarbell SE (2019). Guidelines on management of cyclic vomiting syndrome in adults by the American Neurogastroenterology and Motility Society and the Cyclic Vomiting Syndrome Association. Neurogastroenterol Motil.

[5] Hayes WJ, VanGilder D, Berendse J, Lemon MD, Kappes JA (2018). Cyclic vomiting syndrome: diagnostic approach and current management strategies. Clin Exp Gastroenterol.

[6] Fitzpatrick E, Bourke B, Drumm B, Rowland M (2007). Outcome for children with cyclical vomiting syndrome. Arch Dis Child.

[7] Dignan F, Symon DN, AbuArafeh I, Russell G (2001). The prognosis of cyclical vomiting syndrome. Arch Dis Child.

[8] Fleisher DR, Gornowicz B, Adams K, Burch R, Feldman EJ (2005). Cyclic Vomiting Syndrome in 41 adults: the illness, the patients, and problems of management. BMC Med.

[9] Cooper CJ, Said S, Bizet J, Alkahateeb H, Sarosiek I, McCallum RW (2014). Rapid or normal gastric emptying as new supportive criteria for diagnosing cyclic vomiting syndrome in adults. Med Sci Monit.

[10] Lee LY, Abbott L, Mahlangu B, Moodie SJ, Anderson S (2012). The management of cyclic vomiting syndrome: a systematic review. Eur J Gastroenterol Hepatol.

[11] Shin YK, Kwon JG, Kim KY (2010). A case of cyclic vomiting syndrome responding to gonadotropin-releasing hormone analogue. J Neurogastroenterol Motil.

[12] Saligram S, Bielefeldt K (2014). The two sides of opioids in cyclical vomiting syndrome. N Am J Med Sci.

[13] Zeevenhooven J, Koppen IJ, Benninga MA (2017). The new Rome IV criteria for functional gastrointestinal disorders in infants and toddlers. Pediatr Gastroenterol Hepatol Nutr.

